# Transglutaminase Type 2 regulates the Wnt/β-catenin pathway in vertebrates

**DOI:** 10.1038/s41419-021-03485-2

**Published:** 2021-03-05

**Authors:** Federica Rossin, Roberto Costa, Matteo Bordi, Manuela D’Eletto, Luca Occhigrossi, Maria Grazia Farrace, Nickolai Barlev, Fabiola Ciccosanti, Silvia Muccioli, Leonardo Chieregato, Ildiko Szabo, Gian Maria Fimia, Mauro Piacentini, Luigi Leanza

**Affiliations:** 1 Department of Biology, University of Rome ‘Tor Vergata’, Rome, Italy; 2 Department of Biology, University of Padova, Padova, Italy; 3 Department of Oncohaematology and Cellular and Gene Therapy, IRCSS Bambino Gesù Children’s Hospital, Rome, Italy; 4 Institute of Cytology, Saint-Petersburg, Russia; 5 MIPT, Dolgoprudny, Moscow region, Russia; 6 National Institute for Infectious Diseases IRCCS ‘Lazzaro Spallanzani’, Rome, Italy; 7 Department of Molecular Medicine, University of Rome “La Sapienza”, Rome, Italy

**Keywords:** Biological sciences, Developmental biology

## Abstract

TG2 is a multifunctional enzyme involved in several cellular processes and has emerging as a potential regulator of gene expression. In this regard, we have recently shown that TG2 is able to activate HSF1, the master transcriptional regulator of the stress‐responsive genes; however, its effect on the overall gene expression remains unclear. To address this point, we analyzed, by RNA-seq, the effect of TG2 on the overall transcriptome as well as we characterized the TG2 interactome in the nucleus. The data obtained from these omics approaches reveal that TG2 markedly influences the overall cellular transcriptome profile and specifically the Wnt and HSF1 pathways. In particular, its ablation leads to a drastic downregulation of many key members of these pathways. Interestingly, we found that key components of the Wnt/β-catenin pathway are also downregulated in cells lacking HSF1, thus confirming that TG2 regulates the HSF1 and this axis controls the Wnt signaling. Mechanistic studies revealed that TG2 can regulate the Wnt pathway by physically interacts with β-catenin and its nuclear interactome includes several proteins known to be involved in the regulation of the Wnt signaling. In order to verify whether this effect is playing a role in vivo, we ablated TG2 in *Danio rerio*. Our data show that the zebrafish lacking TG2 cannot complete the development and their death is associated with an evident downregulation of the Wnt pathway and a defective heat-shock response. Our findings show for the first time that TG2 is essential for the correct embryonal development of lower vertebrates, and its action is mediated by the Wnt/HSF1 axis.

## Introduction

Type 2 transglutaminase (TG2) is the most-studied enzyme belonging to the transglutaminase gene family, enzymes that catalyze Ca^2+^-dependent posttranslational modification of proteins, including protein–protein cross-linking, incorporation of primary amines into proteins, and glutamine deamination^1^. Although initially this enzyme was considered purely cytosolic, it is now accepted that TG2 can be localized in other cellular compartments such as nucleus, mitochondria, endoplasmic reticulum and endolysosomes^2^. This multiple subcellular localization and the identification of its substrates in these compartments clearly suggest that TG2 has multiple roles within the cell. Indeed, the enzyme has been shown to be a multifunctional protein having several well-defined enzymatic activities: GTP binding and hydrolysis, protein disulfide isomerase (PDI), and protein kinase activities^3,4^. TG2, by binding GTP, can participate in the transduction of the intracellular signal as part of the G protein coupled receptors^3^. At the mitochondrial level, TG2’s PDI activity has a key role in the production of ATP^5,6^ and it is important for the formation of disulfide bridges in the ATP synthase complex and other key components of the respiratory chain^7,8^. In accordance with this function, TG2 has recently been shown to localize in the MAMs where it plays key structural and functional roles^9^.

Unlike its enzymatic activities, the significance of TG2’s non-enzymatic regulation of its biologicals actions has recently gained importance highlighting that TG2 mostly acts as a scaffold to bridge various proteins, leading to different functional outcomes. Indeed, non-enzymatic interactions of TG2 seem to play physiological roles in a context-specific manner. Several recent data show that cytoplasmic TG2 under specific physiological conditions translocates to the nucleus where it either crosslinks nuclear proteins or interacts with them non-covalently^10^. The nuclear TG2 represents only a limited percentage of the total enzyme in a cell, however various stimuli largely increase its nuclear translocation. There is increasing evidence indicating the importance of nuclear TG2 in regulating gene expression via posttranslational modification or interaction with transcriptional factors (i.e., E2F1, HSF1, Sp1) and histones, in both physiological and pathological settings^11–14^. In this regard, we have recently shown that TG2, by acting as a PDI in the nucleus, is able to activate HSF1, the master transcriptional regulator of the stress‐responsive genes^13^. In fact, TG2 promotes HSF1 trimerization in stressed cells, thereby stimulating specific transactivation of its target genes.

Prompted by these findings, in this study we analyzed the effect of TG2 on the modulation of gene expression by defining its nuclear interactome and its effects on the cellular mRNA profile. In particular, our data clearly show that TG2 modulates gene expression by regulating the Wnt/β-catenin axis. Intriguingly, we also demonstrated that in zebrafish (*Danio rerio*) the TG2-dependent impairment of the Wnt/β-catenin pathway leads to a defective embryonal development. These findings provide a new perspective in our understanding of this enzyme and its involvement in the major human diseases, given the prominent role of Wnt/β-catenin pathway in numerous pathologies^15^.

## Results

### TG2 affects the expression profile and regulates the HS response

**Fig. 1:**
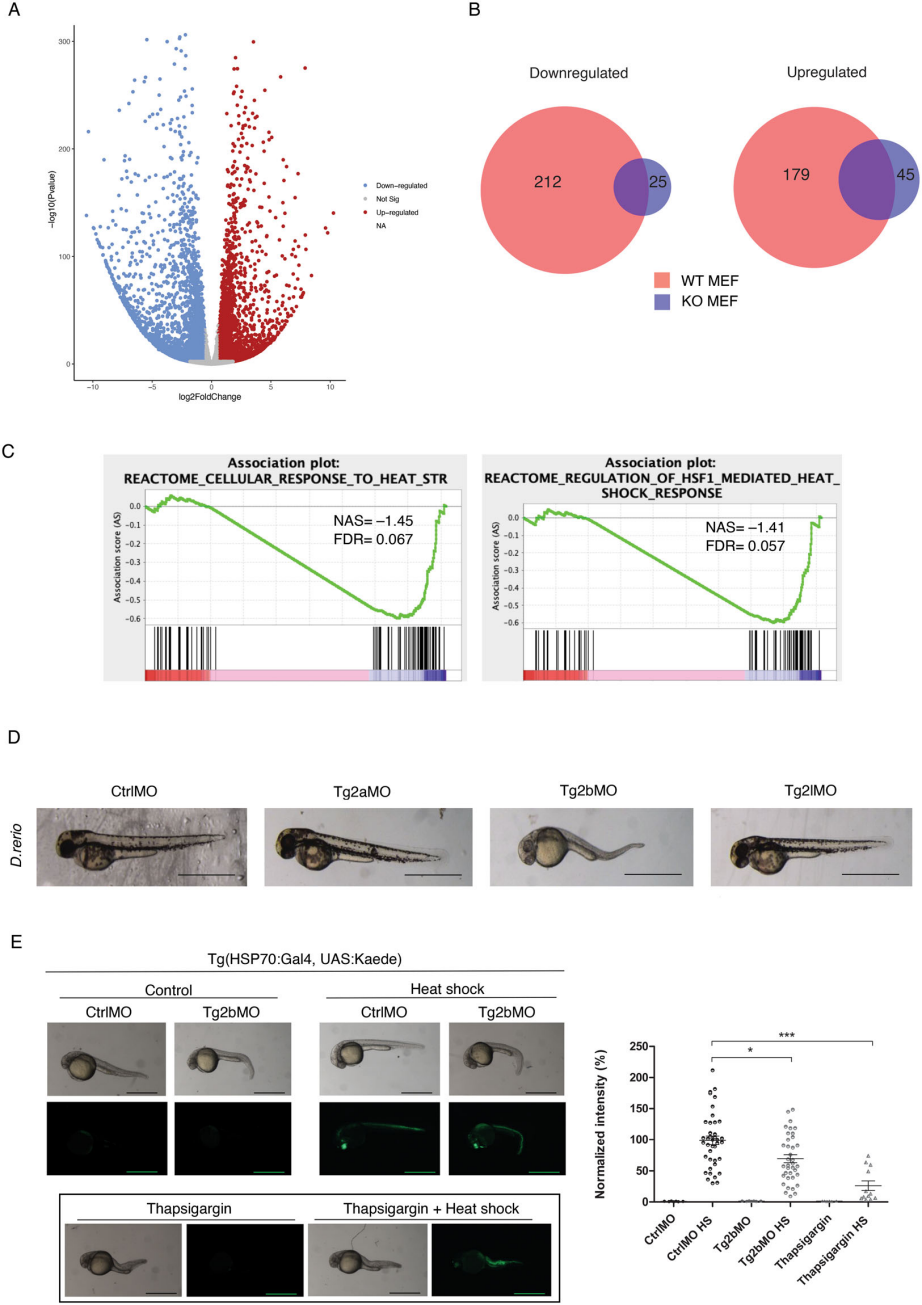
TG2 affects the expression profile and regulates the HS response. **A** Volcano plots for whole transcriptomes of differentially expressed genes between KO and WT MEFs. **B** Venn diagrams illustrating the overlap of the differentially expressed genes (down- or upregulated genes, FC ≤ −1,5 or ≥1,5, *p*val_adj≤0.05) between KO and WT MEFs, under heat shock. **C** Gene set enrichment analysis barcode plot of transcriptomic changes of “Cellular response to heat stress” and “regulation of HSF1 mediated heat shock” (from Reactome database) in KO MEFs compared with WT MEFs. Red, blue, and pink shaded rectangles mark upregulated, downregulated, and genes that are unchanged, respectively. **D** Transient knockdown of the putative TG2 paralogous was performed with three independent morpholinos against zTg2a, zTg2b, and zTg2l; a standard control morpholino was also used. Bright-field images of representative morphants are reported. (*n* = 50). Bars: 1 mm. **E** Double transgenic Tg(-1.5hsp70l:GAL4^(+/−)^; UAS:Kaede^(+/−)^) were injected at one-cell stage either with Tg2bMO or CtrlMO, heat-shock inductions were performed at 24 hpf by incubation of larvae for 1 h at 37 °C, whereas images were acquired between 29 and 30 hpf. Heat-shock protein activation was followed by an increase in the fluorescence. Bright-field and epifluorescent images are reported. The images are representative of different experiments. GFP epifluorescence quantification is reported in the graph on the right. Dots represent treated embryos (*n* of embryos >40). Thapsigargin was used as a positive control. (*n* = 3; means±SEM; *p < 0.05; ***p < 0.001). Bars: 1 mm.

To better understand TG2’s role in the regulation of gene expression, we compared the transcriptome profile of mouse embryonic fibroblasts, derived from WT and KO mice (WT and KO MEFs), by messenger RNA sequencing (RNA-seq). RNA-seq analysis disclosed that TG2 ablation has a high impact on the transcriptome profile of the cells, indeed, a total of 2533 genes were downregulated (fold change ≤ −1.5) and 2206 were upregulated (fold change ≥ 1.5) (Fig. 1a). A Gene Ontology analysis (divided in cellular components, molecular function, and biological process), performed on the downregulated genes, revealed that mostly of these genes were enriched in clusters related to cytoskeleton, actin regulation and extracellular matrix regulation (Supplementary Fig. 1a, b).

Notably, we recently demonstrated that TG2 interacts and activates HSF1, a master transcriptional factor of the heat-shock (HS) genes, thus we asked if TG2 could also affect the expression of genes involved in the HS response^13^. To this aim, we compared the transcriptome of WT and KO MEFs exposed to 20 min of heat shock at 42 °C. We found that the absence of TG2 drastically altered the cellular response to heat shock: the number of upregulated or downregulated genes in KO cells were 45 and 25 compared with 179 and 212, respectively, in WT cells (Fig. 1b).

Moreover, we applied Gene set enrichment analysis that was done on heat-shocked KO MEFs vs heat-shocked wt cells, using Reactome pathway lists “cellular response to heath stress” and “regulation of HSF1 mediated heat-shock response”. Both these lists were significantly less enriched in KO cells (Fig. 1c), meaning a failure in the response to the HS in cells lacking TG2, further confirming the TG2-dependent activation of HSF1.

To corroborate our results, we investigated if TG2 could affect the HS response also in vivo. To this aim, we tested HSP70 response in single TG2 knockdown and in double transgenic Tg(-1.5hsp70l:GAL4(+/−); UAS:Kaede(+/−)) zebrafish. We produced three independent KnockDown (KD) zebrafish lines using antisense morpholinos, since zebrafish harbors three putative zTg2 protein-encoding genes (zTg2a, zTg2b, and zTg2l). Variants A and B encode for the natural proteins (zTg2a and zTg2b)^16,17^, whereas variant L was hypothesized and identified only by sequence prediction. All the zTG2s share sequence homology with the human TG2 protein (NP_001310247.1) as well as the murine one (NP_33399.1), as shown by the phylogenetic tree (Supplementary Fig. 2a). However, among the three zebrafish proteins probably owing to zebrafish gene duplication^18^, the most similar to the human TG2 is the zTg2b.

After 24 h of fertilization (hpf), Tg2aMO and Tg2lMO did not display any significant alteration in phenotype (Fig. 1d). Similarly, no evident morphological changes were observed even at later stages of fish development, at increasing doses of morpholino up to 12 ng per embryo injected. These observations clearly suggest that these transcripts are either not essential during embryonic stages or they are not expressed yet at a given developmental stage. Conversely, in Tg2bMO zebrafish morphants, downregulation of this isoform led to a severe developmental impairment starting from gastrulation stage (Fig. 1d). Around the 40% of morphants died within 48 h, whereas <5% died after the injection with the control morpholino, further confirming that zTg2b transcripts are essential during embryonic development. Moreover, zTg2b KD led to an evident defect in pigmentation completely original for TG2-deficient models (Supplementary Fig. 2b).

Next, we asked whether the observed strong phenotype was linked to a change in HSP70 expression. As expected, in the KD Tg2bMO model, the lack of TG2 partially but significantly impaired HSP70 expression (Fig. 1e), suggesting that heat-shock/UPR response is defective or delayed when compared to control siblings. Thapsigargin, a well-known SERCA inhibitor, was used as control, since thapsigargin blocks the induction of heat-shock response by provoking a generalized impairment in ER homeostasis^19^ (Fig. 1e).

### Loss of TG2 modifies Wnt pathway

**Fig. 2:**
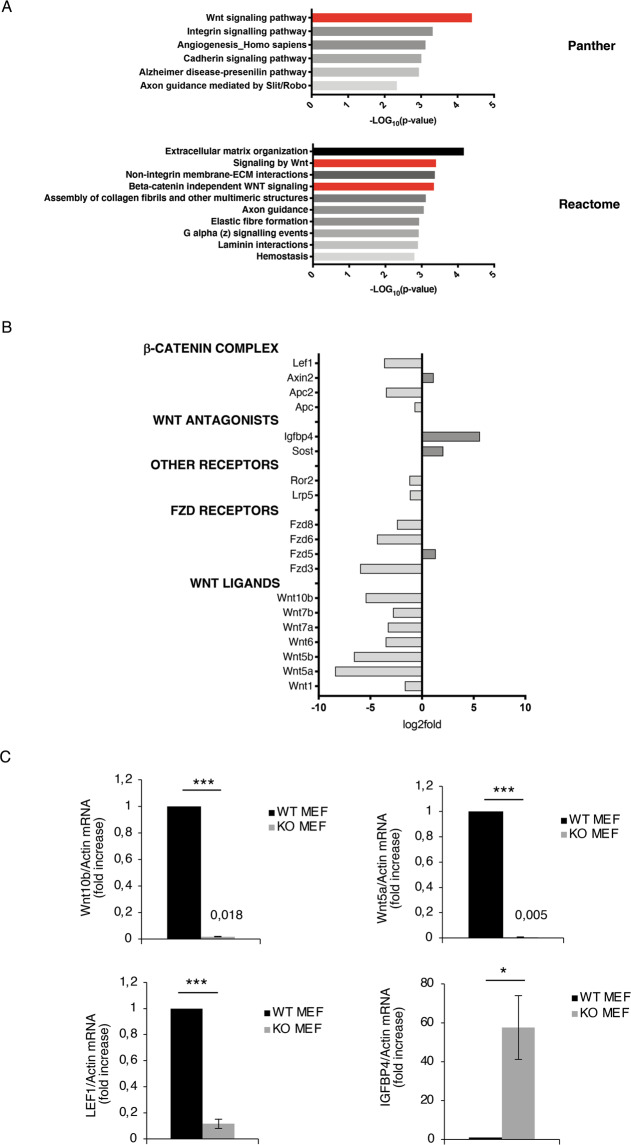
Loss of TG2 modifies Wnt pathway. **A** Pathway enrichment analyses (expressed as −LOG10(*p* value)) using Panther or Reactome database of the significantly downregulated genes in KO MEFs compared with WT. **B** Plot of RNA expression (expressed as Log2 fold changes) of selected genes involved in β-catenin complex and Wnt pathway, comparing KO MEFs versus WT. **C** Wnt10b, Wnt5a, LEF1, and IGFBP4 mRNA levels, quantified by qPCR, in WT and KO MEFs. (*p < 0.05; ***p < 0.001).

Interestingly, enrichment analysis of RNA-seq data, based on annotation terms from Panther and Reactome, identified that Wnt pathway was the most impaired functional pathway (Fig. 2a). In detail, comparative analysis of control versus KO cells showed a global inhibition of many representative Wnt-related genes, including Wnt ligands, receptors, antagonists, and β-catenin interactors (Fig. 2b).

**Fig. 3:**
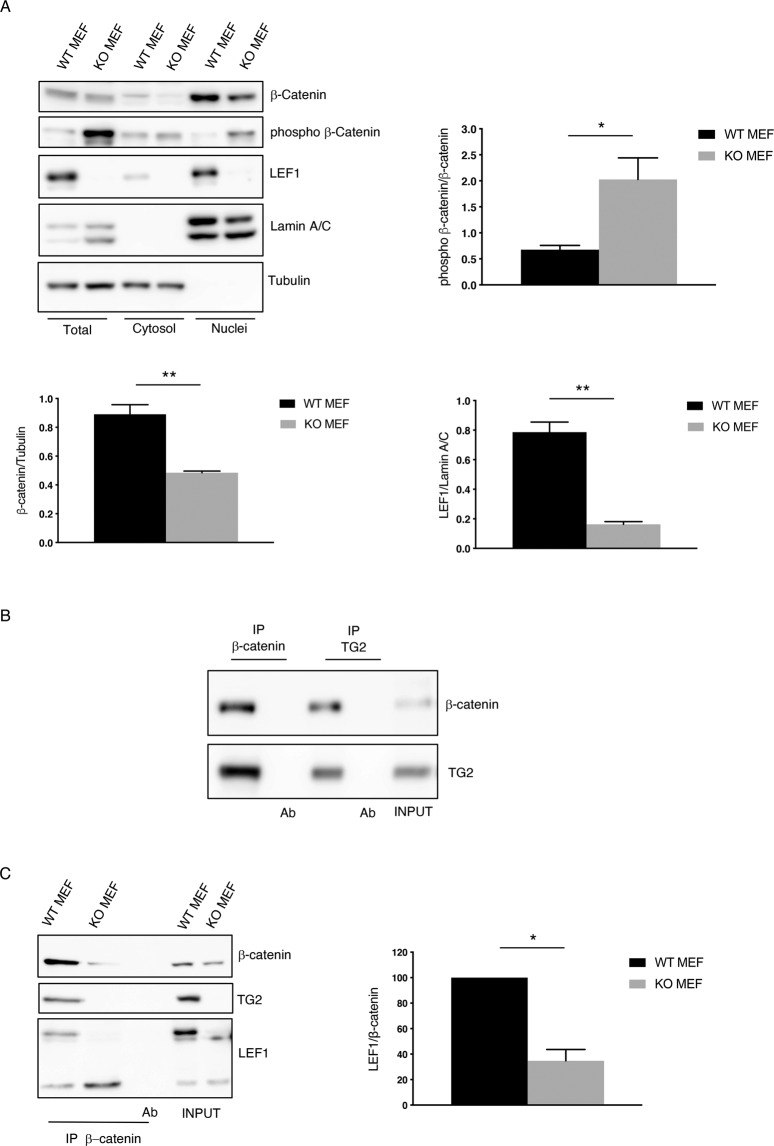
TG2 regulates β-catenin transcriptional activity. **A** Representative western blot and densitometric analysis of β-catenin, its inhibitory phosphorylation, and LEF1 in total, cytosolic and nuclear fractions of WT and KO MEFs. Tubulin was used as loading control for cytosolic extract. Lamin A/C was used as loading control for nuclear extract. (*n* = 3; means±SEM; *p < 0.05; **p < 0.01). **B** Nuclear extract from WT MEFs was subjected to immunoprecipitation for β-catenin and TG2. Immuno- and co-immunoprecipitated proteins were separated by SDS-PAGE and blotted using the indicated antibodies. Input, total cell lysate was used as protein control. **C** Nuclear extracts, obtained from WT and KO MEFs, were subjected to immunoprecipitation for β-catenin. Immuno- and co-immunoprecipitated proteins were separated by SDS-PAGE and blotted using the indicated antibodies. Input, total cell lysate was used as protein control. Densitometric analysis of the ratio of co-immunoprecipitated LEF1/β-catenin. (*n* = 3; means±SEM; *p < 0.05).

RNA-seq data indicate that in the absence of TG2, the Wnt signaling is compromised suggesting a downregulation of the pathway. To confirm these results, we analyzed by qPCR the expression of some Wnt-related genes, such as Wnt10b and Wnt5a (Wnt ligands), the main downstream transcriptional factor LEF1 and the Wnt antagonist Igfbp4. The mRNA levels from WT and KO MEFs, showed in Fig. 2c, confirmed that loss of TG2 led to downregulation of Wnt signaling, also corroborated by the analysis of the protein levels (Fig. 3a and Supplementary Fig. 3a).

The above reported results clearly show that TG2 modulates the expression of HSF1-dependent genes as well as the expression of key components of the Wnt/β-catenin pathway. Interestingly, it has been recently demonstrated that also HSF1 promotes Wnt/β-catenin cascade activation^20^ and among its target genes are included Wnt binding proteins^21^. Prompted by these evidences, we isolated MEFs cells from wild type, HSF1 heterozygous and HSF1 knockout mice (WT, HSF1^+/–^, and HSF1^–/–^ MEFs) to evaluate the expression of Wnt10b and LEF1, two genes of the Wnt pathway, found to be the most downregulated ones in absence of TG2. Thus, we measured the mRNA levels by qPCR analysis and we observed decreased expression in cells lacking HSF1 (Supplementary Fig. 3b). These results propose that not only TG2 but also one of its interacting partners, HSF1, is involved in the regulation of Wnt signaling.

### TG2 regulates β-catenin transcriptional activity

To elucidate how TG2 could affect Wnt signaling expression profile, we analyzed TG2 interacting proteins inside the nucleus. To this aim, we performed immunoprecipitation of nuclear TG2 in WT MEFs and the purified protein complex analyzed by Orbitrap mass spectrometry, resulting in the nuclear TG2’s interactome. Interestingly, we found that some of TG2 interacting proteins are transcriptional factors and most of them are involved in the Wnt/β-catenin pathway (Supplementary Fig. 4a), strengthening the concept that TG2 has a role in the regulation/modification of Wnt signaling components.

Based on these outcomes, we investigated the activation state of the Wnt pathway by evaluating the localization and the phosphorylation of β-catenin. Indeed, in the absence of Wnt ligand, cytoplasmic β-catenin is phosphorylated by CK1α and GSK3, which target it for proteasomal degradation. On the contrary, when Wnt ligand binds to Fz receptor, β-catenin phosphorylation is inhibited allowing β-catenin to accumulate into the nucleus where it works as a LEF1 co-activator for inducing Wnt responsive genes. We performed a cytosolic/nuclear fractionation (Fig. 3a) in WT and KO MEFs and we discovered that, in absence of TG2, the decreased β**-**catenin protein levels correlated with increased phosphorylation on serine 33/37, suggesting that the protein is primed for the degradation. Moreover, according to the gene expression analysis, also the LEF1 protein level was lower in the nucleus of cells lacking TG2 in comparison with WT cells.

To evaluate how TG2 could promote β-catenin stabilization, we performed a co-immunoprecipitation of β-catenin and TG2 and we found they interact with each other in the nucleus (Fig. 3b). Prompted by these findings, we investigated whether TG2 could affect the interaction of β-catenin with LEF1, inside the nucleus, necessary to induce Wnt genes expression. To this aim, we immunoprecipitated nuclear β-catenin in WT and KO MEFs and found that cells lacking TG2 showed a decreased interaction with LEF1, confirming a defect in Wnt pathway activation (Fig. 3c). These results suggest that TG2, by binding β-catenin, could prevent its degradation and promote the interaction with key components inside the nucleus leading to the activation of Wnt signaling.

**Fig. 4:**
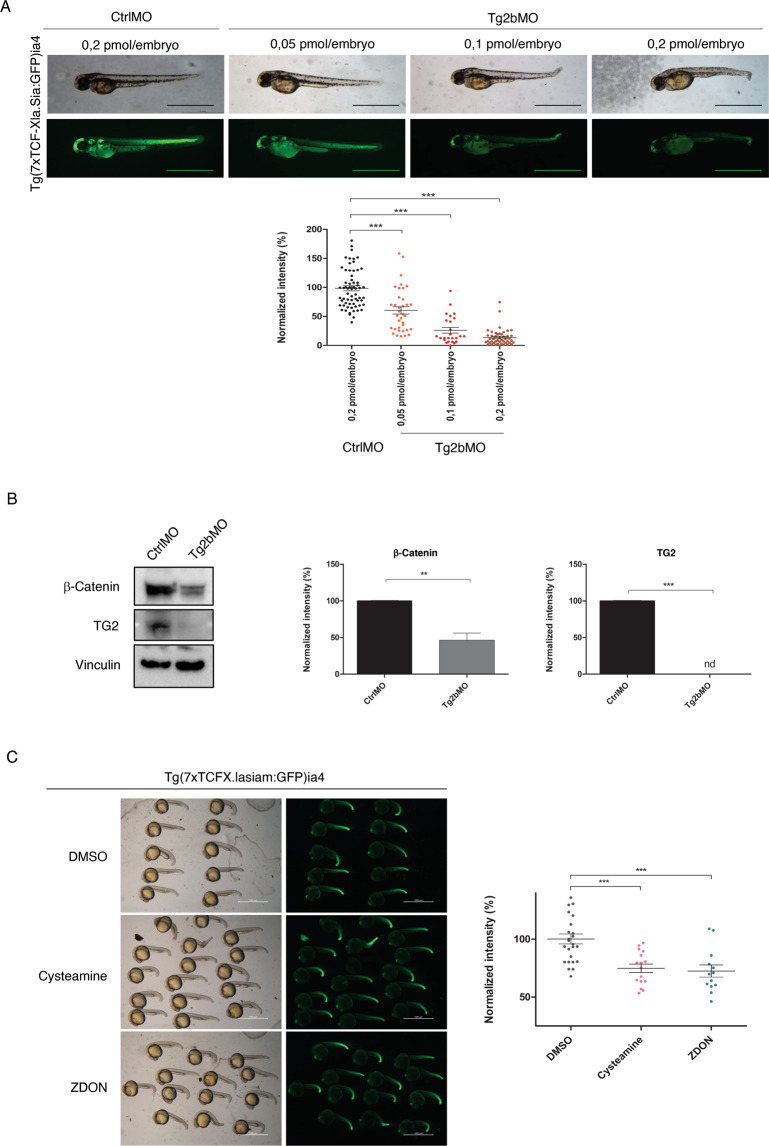
TG2 modulation of Wnt pathway in the zTg2b zebrafish model. **A** Knockdown of the zTg2b was performed in Tg(7xTCFX.lasiam:GFP)ia4 Wnt-dependent reporter fishes by injecting three increasing dosages of morpholinos (0.05, 0.1, and 0.2 pmol). Zebrafish at 48hpf were observed by bight field and epifluorescence microscopy. The images are representative for the groups of animals and the GFP quantification is reported in the graph (*n* = 50; means±SEM; ***p < 0.001). Bars: 1 mm. **B** Zebrafish TG2 protein and zebrafish β-catenin protein from KD and control larvae’s protein extracts were compared by western blot. The quantification is reported in the graph below. The values are indicated as the percentage of the control samples set as 100% (*n* = 3; means±SEM; **p < 0.01; ***p > 0.001). **C** GFP fluorescent zebrafish Tg(7xTCFX.lasiam:EGFP)ia4 Wnt-dependent reporter fishes were treated for 15 h with the following compounds: DMSO 0.2%, 40 μM ZDON, 1 mM Cysteamine. Epifluorescence microscopy images are reported, whereas fluorescence quantification is shown in the graph (*n* = 50; means±SEM; ***p < 0.001). Bars: 1.5 mm.

To corroborate our findings, we also evaluated TG2 modulation of Wnt pathway in vivo in the zTg2b zebrafish model, by using the well-established reporter line Tg(7xTCFX.lasiam:EGFP)^ia4^, which highlights the canonical Wnt^22^. Ablation of zTg2b in Tg(7xTCFX.lasiam:EGFP)^ia4^ repressed Wnt signaling (Fig. 4a). Interestingly, the injection of incrementing doses (0.05, 0.1, and 0.2 pmol) of antisense morpholino resulted in progressive alterations in morphology and body shape correlated to a progressive reduction of Wnt-dependent fluorescence. As a control of the specificity for zTg2b KD on Wnt signaling, three further developmental pathways were studied in vivo: (I) glial fibrillary acidic protein (GFAP) signaling, which highlights neural development, was not reduced by zTg2b KD, suggesting that Wnt reduction is not mediated by general developmental delay; (II) the erythroid transcription factor Gata, which marks blood cells progenitors, is upregulated when compared with control. These results were in agreement with previous observations showing that Wnt ablation by chemical compounds and/or morpholinos improves Gata1 signaling;^23^ (III) the vascular marker *Kdrl* (also known as *egfr1* in *Homo sapiens*), which is normally positively regulated by Wnt signaling in *D. rerio* during vascular development^23^, is lowered when Wnt signaling is impaired (Supplementary Fig. 4b).

Wnt downregulation, shown as a decrease in β-catenin protein, was further confirmed by western blotting upon zTg2b ablation (Fig. 4b). Finally, TG2 mediated regulation of Wnt signaling in vivo has also been demonstrated by treatment with TG2 inhibitors. Indeed, Cysteamine or ZDON, well-known TG2 modulators^1,24,25^, can significantly reduce Wnt-dependent GFP fluorescence in Tg(7xTCFX.lasiam:EGFP)^ia4^ zebrafish reporter line (Fig. 4c).

Taken together, these results clearly demonstrate that TG2 is essential to sustain Wnt signaling in vitro and in vivo, by modulating Wnt-dependent transcriptional activity, e.g., promoting β-catenin stabilization and Wnt target genes expression (e.g., LEF1).

## Discussion

TG2 is a peculiar multifunctional enzyme which, in addition to catalyze the canonical Ca^2+^-dependent posttranslational modifications of proteins, similarly to the other members of the transglutaminase family, has been shown to catalyze other biochemical activities such as serine/threonine kinase and PDI^26^ Moreover, TG2 has been reported to play structural non-enzymatic protein–protein interactions in various cellular compartments. Indeed, TG2 acts as G protein in transmembrane signaling linked to various protein receptors or binds to GRP75, a key bridging molecule of the mitochondrial/endoplasmic reticulum interaction sites, thus controlling the intracellular Ca^2+^ homeostasis^2^. A number of data have pointed out the involvement of this complex protein in the regulation of gene expression; however, a systematic investigation of this potential function has never been clearly carried out. In keeping with these notions, it has been shown that the primary sequence of TG2 contains two putative bipartite nuclear localization signals NLS and that the enzyme may be actively transported into the nucleus via binding to an importin‐α3/Qip‐1 family protein^27^. Kojima’s group demonstrated that the enzyme is playing a key role in hepatocyte apoptosis induced by both ethanol and Fas by the selective cross-linking inactivation of the transcription factor Sp1, resulting in reduced expression of growth factor receptors such as c-Met^28^. Moreover, we have shown that upon cellular stress, TG2 translocates in the nucleus where, by its PDI activity, it oligomerizes the HSF1 transcription factor, the master regulator of transcription during cellular heat-shock response^13^. Recently, it has been also demonstrated that TG2 can serotonylate histone H3 tri-methylated lysine 4 (H3K4me3)-marked nucleosomes, resulting in the presence of combinatorial H3K4me3Q5ser in vivo^11^. Interestingly, the TG2-dependent transcriptional regulation of gene expression seems to be evolutionary conserved, in fact, in *Drosophila*, the TG-catalyzed Relish cross-linking suppressed the immune deficiency pathway that enables immune tolerance against commensal microbes^29^.

Prompted by these evidences, in this study we investigated the role of TG2 in the regulation of gene expression. To this aim, we have carried out the whole-RNA-seq analysis comparing wild-type and TG2 knockout MEFs under normal conditions as well as after heat shock. The data clearly evidenced the profound effect of TG2 on gene expression. Indeed, a total of 2533 and 2206 genes were, respectively, downregulated and upregulated in cells lacking TG2, highlighting the high impact of the enzyme on the transcriptome profile. We found that many key pathways are modulated by TG2, confirming its role in fundamental biological processes such as extracellular matrix organization, G protein function, and neurodegenerative disorders such as Alzheimer disease^28^. In addition, enrichment analysis of RNA-seq data revealed that cholesterol biosynthesis is the most-enriched gene set together with Ppar-γ pathway, confirming previous data reporting intracellular fat accumulation in cells lacking TG2 (ref. ^30^). Interestingly, in the absence of TG2, the heat-shock response was dramatically affected both in vitro and in zebrafish, thus confirming the evolutionary conserved role of TG2 in the regulation of HSF1 (ref. ^13^). Noteworthy, our analysis clearly identified the Wnt/β-catenin signaling pathway as the most affected in the absence of TG2. These findings are also corroborated by the identification of the TG2 nuclear-interacting proteins. Indeed, we found that TG2 interacts with several important transcriptional factors, which have been shown to play a key regulatory function in the modulation of the Wnt/β-catenin pathway. Future studies should define whether these proteins are substrates that can be post-translationally modified by TG2 as well as the consequent downstream cellular events.

In order to confirm this effect in vivo, we ablated transglutaminases in zebrafish and we analyzed the effect on the expression of the Wnt pathway. The data clearly evidenced, also in lower vertebrates, the drastic effect elicited by the TG2 ablation on the Wnt pathway. Interestingly, the same effect on the Wnt signaling was obtained by inhibiting the enzymatic activity of TG2. This result highlights the importance of the catalytic activity of the enzyme for the induction of the Wnt pathway and for the proper development of zebrafish. Indeed, it is important to note that in zebrafish three zTG isoforms exist but interestingly only the zTg2b downregulation determines a severe developmental impairment starting from gastrulation stage. In fact, ~40% of morphants died within 48 h, indicating that zTg2b transcripts are essential during embryonic development. TG2 KD in *D. rerio* also showed an evident defect in pigmentation, which is usually observed in Wnt deficient animals, but it is completely original for TG2-deficient models. Indeed, Wnt signaling is a critical player in epidermal melanophores (also known as melanocytes in mammals)^31,32^. These data are particularly important because the knockdown of TG2 in mammals does not produce any evident developmental phenotype, suggesting that the other TGs isoforms could compensate for the absence of TG2 during embryonal development.

In conclusion, this study demonstrates the role of TG2 on gene expression and for the first time highlights its fundamental role in embryonal development in vertebrates. The identification of the TG2 modulation of the Wnt/β-catenin is also important to explain the involvement of the enzyme in pathological settings such as cancer and diabetes.

## Materials and methods

### Cells

WT and KO MEFs were obtained from C57BL/6 mice either wild type or knockout for TG2. HSF1^+/–^ and HSF1^–/–^ MEFs were obtained from C57BL/6 mice heterozygous and knockout for HSF1. Fibroblasts were isolated by trypsinization of embryos at E14. The dissociated cells were plated and grown to near-confluence and were passed every 3 or 4 days until spontaneous immortalization occurred. MEF cells were cultured in Dulbecco’s modified Eagle’s medium (Lonza) supplemented with 10% fetal bovine serum, 100 μg/ml streptomycin, and 100 units/ml penicillin, at 37 °C and 5% CO_2_ in a humidified atmosphere. Mycoplasma contamination was tested in all cell lines. To induce HS cells were placed in a water bath at 42 °C for 20 min.

### RNA sequencing

Next-generation sequencing experiments were performed by Genomix4life S.R.L. (Baronissi, Salerno, Italy). RNA was isolated and concentration in each sample (three samples for each condition) was assayed with a ND-1000 spectrophotometer (NanoDrop) and its quality assessed with the TapeStation 4200 (Agilent Technologies). Indexed libraries were prepared from 500 ng/ea purified RNA with TruSeq Stranded mRNA Sample Prep Kit (Illumina) according to the manufacturer’s instructions. Libraries were quantified using the TapeStation 4200 (Agilent Technologies) and and Qubit fluorometer (Invitrogen Co.), then pooled such that each index-tagged sample was present in equimolar amounts, with final concentration of the pooled samples of 2 nM. The pooled samples were subject to cluster generation and sequencing using an Illumina NextSeq 500 System (Illumina) in a 2 × 75 paired-end format at a final concentration of 1.8 pmol.

The raw sequence files generated (.fastq files) underwent quality control analysis using FastQC (http://www.bioinformatics.babraham.ac.uk/projects/fastqc) and the quality checked reads were trimmed with cutadapt^33^ v.1.10 and then aligned to the mouse genome (GRCm38) using STAR v.2.5.2^34^, with standard parameters. Differentially expressed mRNAs were identified using DESeq2 v.1.12^35^. First, gene annotation was obtained for all known genes in the human genome, as provided by GenCode (GRCm38.p6 release 17). Using the reads mapped to the genome, we calculated the number of reads mapping to each transcript with HTSeq-count v.0.6.1^36^. These raw read counts were then used as input to DESeq2 for calculation of normalized signal for each transcript in the samples, and differential expression was reported as fold change along with associated adjusted *p* values (computed according to Benjamini–Hochberg). The data files have been deposited in the Gene Expression Omnibus (GEO) database under the accession number GSE 162071. The Gene ontology and pathway enrichment analysis were performed by using Enrichr online tool^37^. The Volcano plot was generated using Galaxy platform (http://galaxyproject.org). The gene set enrichment tool Enrichr^37^ was used to perform functional annotation analysis of REACTOME pathways. Gene Set Association Analysis (GSAA)^36^ of the expression data was used to assess enrichment of selected gene lists, indicated, respectively, in the figure legend, derived from REACTOME Pathway Database. We used the GSAASeqSP tool, a Java-based desktop application (software GSAA 2.0), according to the manufacturer’s instructions^38^, and we applied the default setting, choosing Signal2Noise_log2Ratio for differential expression analysis of individual genes.

### Quantitative RT-PCR

Cells were lysed in Trizol reagent (Invitrogen, Carlsbad, CA) and total RNA was extracted using Direct-Zol™ RNA MiniPrep Plus according to the manufacturer’s instructions. In all, 1 μg of RNA was retro‐transcribed using SensiFAST™ cDNA Synthesis Kit (Bioline) and used in quantitative RT-PCR (qPCR) experiment, using SensiFAST™ SYBR Hi-ROX Kit (Bioline) following manufacturer’s instructions. Thermocycling consisted of an initial polymerase activation step at 98 °C for 5 min, and amplification was performed with 35 cycles of 95 °C for 15 s, 68 °C for 10 s, and 72 °C for 20 s with data acquisition at this stage and the reaction finished by the built‐in melt curve. The relative amounts of mRNA were calculated by using the comparative Ctmethod.

Actin: 5′GGCTGTATTCCCCTCCATCG3′, 5′CCAGTTGGTAACAATGCCATGT3′

LEF1: 5′AAATGGGTCCCTTTCTCCAC3′, 5′TCGTCGCTGTAGGTGATGAG3′

Wnt10b: 5′ACGACATGGACTTCGGAGAGAAGT3′, 5′CATTCTCGCCTGGATGTCCC3′

Wnt5a: 5′TGAAGCAGGCCGTAGGAC3′, 5′AGCCAGCACGTCTTGAGG3′

IGFBP4: 5′TGAGAGCGAACATCCCAACAA3′, 5′TGTCCCCACGATCTTCATCTT3′

### Co-immunoprecipitation

Cells were lysed in a buffer containing 150 mM NaCl, 50 mM Tris–HCl pH 7.5, 2 mM EDTA, 2% NP‐40 and freshly added protease inhibitor cocktail. An amount of 2 mg of proteins from cell lysates were subjected to immunoprecipitation using 4 μg of specific antibodies in combination with 30 μl of Dynabeads™ Protein G (Invitrogen), according to the manufacturer’s instructions. LDS sample buffer 2× (Life Technologies) containing 2.86 M 2‐mercaptoethanol (Sigma‐Aldrich) was added to beads, and samples were boiled at 95 °C for 10 min. Supernatants were analyzed by western blot.

### Sample preparation and LC-MS/MS analysis

The first and second elutions of single immunoprecipitation were pooled and boiled at 95 °C; disulfide bonds were reduced and alckylated, respectively, with 10 mM dithiothreitol, 30 min at 56 °C, and 55 mM iodoacetamide, 20 min at room temperature in darkness. After precipitation with ethanol 100%, the samples were resuspended in ammonium bicarbonate 50 mM, 2 M urea and were digested by trypsin (0.2 μg/sample) at 37 °C overnight. Then were purified through the filter of a Zip-Tip (C-18 Resin, Millipore) and eluted from the Zip-Tip with 80% acetonitrile and 0.1% TFA. To eliminate the excess of acetonitrile, speedvac was used for 3 min and the peptides were resuspended in 10 μl of 2.5% acetonitrile, 0.1% TFA, and 10 μl 0.1% formic acid.

### Mass spectrometry analysis and protein identification

The peptide mixture were analyzed by ultra-high performance liquid chromatography coupled with high resolution mass spectrometry using Thermo Scientific Q Exactive Plus Orbitrap; in particular, the peptides were separated by nano liquid chromatography (UltiMate 3000 RSLC nano-LC system, Thermo Fisher Scientific, Waltham, MA, USA), loaded onto a 75 μm C18column (ES800—Thermo Fisher Scientific, Waltham, MA, USA), using a 100 min multistep gradient elution (from 4–90% eluent B with a constant flow of 0.3 μL/min), and were analyzed by Exactive Q plus mass spectrometer (Thermo Fisher Scientific). The raw data from the mass spectrometric analysis was processed using the MaxQuant sofware v.1.5.5.1.

### Western blot analysis

MEFs cells were rinsed in ice‐cold PBS and collected in lysis buffer containing 20 mM Tris–HCl pH 7.4, 150 mM NaCl, and 1% Triton X‐100 with protease inhibitor cocktail. Nuclear and cytosolic extracts were obtained using the NE‐PER Nuclear and Cytoplasmic Extraction Kit (Thermo Scientific). Protein concentrations were determined by the Bradford assay, using bovine serum albumin as a standard. Aliquots of total protein extracts from cells after different treatments were resolved on sodium dodecyl sulfate (SDS)–polyacrylamide gel and transferred to a nitrocellulose membrane. Blots were blocked in 5% non‐fat dry milk in T‐PBS (PBS + 0.05% Tween‐20) for 1 h at room temperature and then incubated overnight with the described antibodies. The membranes were incubated with enzyme horseradish peroxidase (HRP)‐conjugated secondary antibody for 1 h at room temperature, and the signal was detected by Immobilon® Western (Millipore).

### Antibodies

Anti LEF1 (sc374522, Santa Cruz); anti β−catenin (ab32572, AbCam); anti phospho β−catenin (9561, Cell Signaling); anti TG2 (3557, Cell signaling); anti TG2 (sc71632, Santa Cruz); anti Lamin A/C (sc-6215, Santa Cruz); anti Tubulin (T-4026, Sigma); anti Wnt5a (MAB645, R&D); anti Wnt10b (PA5-20530, Invitrogen).

### Multiple alignment of natural proteins

The phylogenetic analysis of TG2 proteins has been performed by Neighbor-Joining method, using ClustalX2 and TreeView software solutions. The input sequences were already reported. Accession numbers are reported in the “rooted cladogram” tree.

### Zebrafish reporter lines

WT zebrafish were from the Tübingen (Tü) or AB strains. All transgenic lines were collected from original laboratories, which developed the lines and are currently stabled at the zebrafish facility of University. Fish housing was carried out at 28.5 °C according to standard rules and procedures (https://zfin.org). Tg(7xTCFX.lasiam:GFP)ia4 contains a gfp gene driven by a tcf/lef responding promoter. This line reports Wnt signaling activity in vivo. Tg(gfap:eGFP)mi2001 carries an EGFP reporter transgene under the control of GFAP promoter region^39^. Tg(Kdrl:mCherry)uto2 expresses a red fluorescent protein in vascular cells^40^. Tg(gata1:dsRed)sd2 carries the dsRed gene under control of a 7-kb fragment derived from the gata1 promoter^41^.

Zebrafish were also treated with TG2 inhibitors Cysteamine (1 mM) or ZDON (40 μM) in can significantly reduce GFP fluorescence by O/N treatment (12–30 hpf) if compared with the dimethyl sulfoxide-treated control.

All animal manipulation procedures were conducted according to the Local Ethical Committee at the University of Padua and National Agency (Italian Ministry of Health) (Italian Ministry of Health Authorization number 407/2015-PR), and with the supervision of the Central Veterinary Service of the University of Padova (in compliance with Italian Law DL 116/92 and further modifications, embodying UE directive 86/609).

### Morpholino injection

Custom Morpholinos were purchased by Genetools LLC. The sequence used are the follow:

Tg2aMO: (5′-CTCAATTTCCACCACTCTCTCCATG-3′);

Tg2bMO: (5′-CCGATGTCCAGAGCCATGTTTATAA-3′);

Tg2lMO: (5′-TGATGGATTTAGCTTGACAAGTCGT-3′).

All morpholinos affect translational start of zebrafish TG2 paralogous genes. Standard Ctrl Morpholino (CtrlMO) was also purchased by Genetools LLC. Microinjection was performed on randomly separated sibling embryos at one-cell stage, adding ≈12 ng/embryos of morpholino. Morpholinos impeded either the translation of zTg2a, or zTg2b, or zTg2l transcripts, blocking both maternal and zygotic forms. Chorions were manually removed at 24 hpf (hpf: hours post fertilization) and images were acquired either at 30 or 48hpf, crude proteins extract were also obtained after image acquisition. zTg2b KD was performed by the injection of 0.05, or 0.1, or 0.2 pmol of antisense morpholino for embryo.

### Microscopy and image acquisition

Zebrafish embryos expressing fluorescent proteins were analyzed using a Leica M165FC epifluorescent microscope. All images were acquired with a Nikon DS-F12 digital camera. All images were acquired with the same exposure parameters and processed in silico with Gimp 2.0 and Adobe Photoshop. A single-embryo representative for the whole population was reported in each picture.

### Western blot with zebrafish samples

Fish larvae at 2dpf (dpf: days post fertilization) were lysed according to the protocol proposed earlier^42^. Lysis buffer were supplemented with protease inhibitors cocktail (Roche Diagnostics, Mannheim, Germany) and Phosphatase Inhibitor Cocktails 2 and 3 (Sigma Aldrich, Milan, Italy). Samples homogenization were performed by a glass dounce. The supernatant was collected, and protein concentration was determined by the Bradford method. Extracted proteins were supplemented with 4× sample buffer, heated at 80 °C for 5 min and run onto precast SDS-PAGE (Thermofisher, Milan, Italy). Proteins were transferred on Immobilon-P membranes (Merk Millipore, Italy) with Biorad turbo semi-dry system (30 min, 25 V) in 25 mM Tris, 192 mM glycine, 10% ethanol (v/v), 0.1% SDS. Membranes were incubated with the following antibodies: anti β-catenin (C7082, Sigma Aldrich); anti Vinculin (AB6039, EMD Millipore); anti TG2 (3557, Cell signaling), anti-mouse or anti-rabbit secondary antibodies conjugated to horseradish peroxidase (A4416, Sigma Aldrich and 074-1516, KPL). All primary antibodies were used at 1:1000 dilution. HRP Chemiluminescence signals were detected by Biorad Chemidoc Image Station.

### Heat-shock experiments in zebrafish

Homozygous Tg(-1.5hsp70l:GAL4) were out-crossed with homozygous Tg(UAS:Kaede), generating heterozygous double carriers, which can remark heat-shocked larvae by green fluorescent like protein accumulation^43^. Double transgenic siblings were injected at one-cell stage either with Tg2bMO or CtrlMO, heat-shock inductions were performed at 24 hpf by incubation of larvae for 1 h at 37 °C, whereas images were acquired between 29 and 30 hpf.

### Statistical analysis

The sample size has been identified, considering the theoretical difference between the means and the theorical size of the standard deviation/s. Animals were randomly associated with the treatments and operator blinding was not done. GraphPad was used for statistical analysis. ImageJ64 software was used for densitometric analysis. Statistical significance was determined using the Student’s *t* test or one‐way analysis of variance test. *P* value smaller than 0.05 (*p* < 0.05) was considered to be significant.

## Supplementary information

Supplementary figures

## Data Availability

The data sets generated and analyzed during the current study are available in the GEO repository, accession number GSE 162071.

## References

[1] Lee CS, Park HH (2017). Structural aspects of transglutaminase 2: functional, structural, and regulatory diversity. Apoptosis.

[2] D’Eletto M, Rossin F, Fedorova O, Farrace MG, Piacentini M (2018). Transglutaminase type 2 in the regulation of proteostasis. Biol. Chem..

[3] Im MJ, Graham RM (1990). A novel guanine nucleotide-binding protein coupled to the alpha 1-adrenergic receptor. I. Identification by photolabeling or membrane and ternary complex preparation. J. Biol. Chem..

[4] Hasegawa G (2003). A novel function of tissue-type transglutaminase: protein disulphide isomerase. Biochem J..

[5] Altuntas S (2014). Type 2 Transglutaminase, mitochondria and Huntington’s disease: menage a trois. Mitochondrion.

[6] Rossin F, D’Eletto M, Farrace MG, Piacentini M (2014). Transglutaminase type A multifunctional protein chaperone?. Mol. Cell Oncol..

[7] Battaglia G (2007). Transglutaminase 2 ablation leads to defective function of mitochondrial respiratory complex i affecting neuronal vulnerability in experimental models of extrapyramidal disorders. J. Neurochem.

[8] Mastroberardino PG (2006). “Tissue” transglutaminase contributes to the formation of disulphide bridges in proteins of mitochondrial respiratory complexes. Biochim. Biophys. Acta.

[9] D’Eletto M (2018). Transglutaminase type 2 regulates ER-mitochondria contact sites by interacting with GRP75. Cell Rep..

[10] Kanchan K, Fuxreiter M, Fésüs L (2015). Physiological, pathological, and structural implications of non-enzymatic protein-protein interactions of the multifunctional human transglutaminase 2. Cell Mol. Life Sci..

[11] Farrelly LA (2019). Histone serotonylation is a permissive modification that enhances TFIID binding to H3K4me3. Nature.

[12] Oliverio S (1997). Tissue transglutaminase-dependent posttranslational modification of the retinoblastoma gene product in promonocytic cells undergoing apoptosis. Mol. Cell Biol..

[13] Rossin F (2018). TG2 regulates the heat‐shock response by the post‐translational modification of HSF1. EMBO Rep..

[14] Tatsukawa H (2009). Role of transglutaminase 2 in liver injury via cross-linking and silencing of transcription factor Sp1. Gastroenterology.

[15] Moon RT, Kohn AD, De Ferrari GV, Kaykas A (2004). WNT and beta-catenin signalling: diseases and therapies. Nat. Rev. Genet..

[16] Housley MP (2014). Translational profiling through biotinylation of tagged ribosomes in zebrafish. Development.

[17] Rauwerda H (2017). Transcriptome dynamics in early zebrafish embryogenesis determined by high-resolution time course analysis of 180 successive, individual zebrafish embryos. BMC Genomics.

[18] Howe K (2013). The zebrafish reference genome sequence and its relationship to the human genome. Nature.

[19] Ron D (2002). Translational control in the endoplasmic reticulum stress response. J. Clin. Invest..

[20] Zhang L (2019). Proteomics analysis of proteins interacting with heat shock factor 1 in squamous cell carcinoma of the cervix. Oncol. Lett..

[21] Kovács D (2019). HSF1Base: a comprehensive database of HSF1 (heat shock factor 1) target genes. Int. J. Mol. Sci..

[22] Moro E (2012). In vivo Wnt signaling tracing through a transgenic biosensor fish reveals novel activity domains. Dev. Biol..

[23] Hübner K (2017). Wnt signaling positively regulates endothelial cell fate specification in the Fli1a-positive progenitor population via Lef1. Dev. Biol..

[24] McConoughey SJ (2010). Inhibition of transglutaminase 2 mitigates transcriptional dysregulation in models of Huntington disease. EMBO Mol. Med.

[25] Palucci I (2017). Transglutaminase type 2 plays a key role in the pathogenesis of Mycobacterium tuberculosis infection. J. Intern. Med..

[26] Ricotta M, Iannuzzi M, De Vivo G (2010). & Gentile V. Physio-pathological roles of transglutaminase-catalyzed reactions. World J. Biol. Chem..

[27] Peng X (1999). Interaction of tissue transglutaminase with nuclear transport protein importin-alpha3. FEBS Lett..

[28] Tatsukawa H, Furutani Y, Hitomi K, Kojima S (2016). Transglutaminase 2 has opposing roles in the regulation of cellular functions as well as cell growth and death. Cell Death Dis..

[29] Shibata T (2013). Transglutaminase-catalyzed protein-protein cross-linking suppresses the activity of the NF-κB-like transcription factor relish. Sci. Signal..

[30] Piacentini M (2018). Non-alcoholic fatty liver disease severity is modulated by transglutaminase type 2. Cell Death Dis..

[31] Vibert L (2017). An ongoing role for Wnt signaling in differentiating melanocytes in vivo. Pigment Cell Melanoma Res..

[32] Sun Q (2018). Dissecting Wnt signaling for melanocyte regulation during wound healing. J. Invest. Dermatol..

[33] Martin M. “Cutadapt removes adapter sequences from high-throughput sequencing reads”. *EMBnet J.* **17**, 1 (2011)

[34] Dobin A (2013). STAR: ultrafast universal RNA-seq aligner. Bioinformatics.

[35] Love MI, Huber W, Anders S (2014). Moderated estimation of fold change and dispersion for RNA-seq data with DESeq2. Genome Biol..

[36] Anders S, Pyl PT, Huber W (2015). HTSeq–a Python framework to work with high-throughput sequencing data. Bioinformatics.

[37] Kuleshov MV (2016). Enrichr: a comprehensive gene set enrichment analysis web server 2016 update. Nucleic Acids Res..

[38] Xiong Q, Mukherjee S, Furey TS (2014). GSAASeqSP: a toolset for gene set association analysis of RNA-Seq data. Sci. Rep..

[39] Jing X, Malicki J (2009). Zebrafish ale oko, an essential determinant of sensory neuron survival and the polarity of retinal radial glia, encodes the p50 subunit of dynactin. Development.

[40] Wang Y (2010). Moesin1 and Ve-cadherin are required in endothelial cells during in vivo tubulogenesis. Development.

[41] Traver D (2003). Transplantation and in vivo imaging of multilineage engraftment in zebrafish bloodless mutants. Nat. Immunol..

[42] Schnabel D, Castillo-Robles J, Lomeli H (2019). Protein purification and western blot detection from single Zebrafish eembryo. Zebrafish.

[43] Ando R, Hama H, Yamamoto-Hino M, Mizuno H, Miyawaki A (2002). An optical marker based on the UV-induced green-to-red photoconversion of a fluorescent protein. PNAS.

