# Supplementary material for: Transglutaminase Type 2 regulates the Wnt/β-catenin pathway in vertebrates
Source: Cell Death Dis. 2021 Mar 5;12(3):249. doi: 10.1038/s41419-021-03485-2 (PMC7935911; doi:10.1038/s41419-021-03485-2)
Supplement: Supplementary file 1 — Supplementary figures [file 41419_2021_3485_MOESM1_ESM.pdf]

A

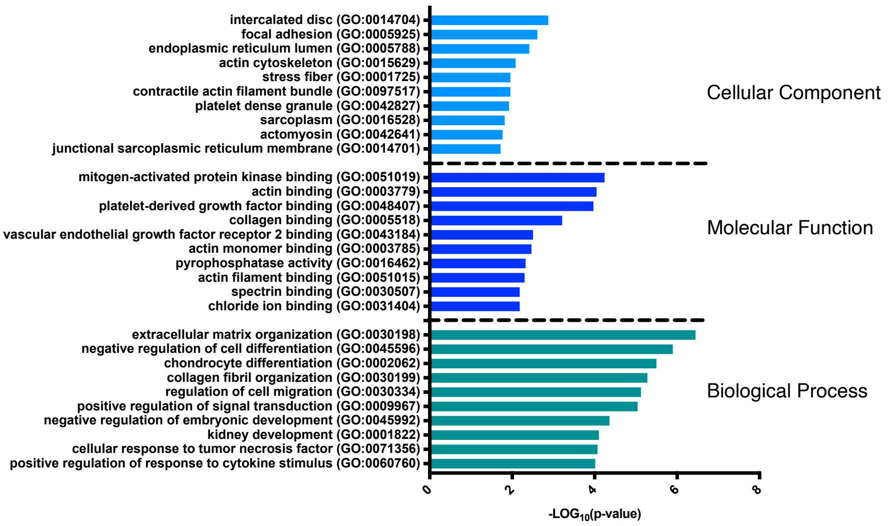

B

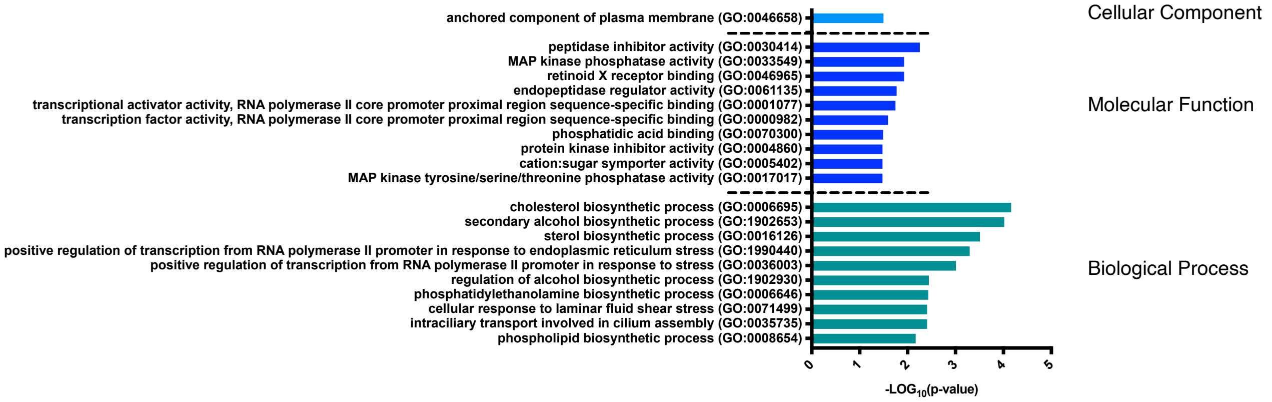

Supplementary Fig. S1 A-B Gene ontology (GO) terms (expressed as -LOG10(p-value)) with the highest significance are shown. GO analysis of transcripts enriched in significantly downregulated (**A**) and upregulated (**B**) genes in KO MEFs compared to WT.

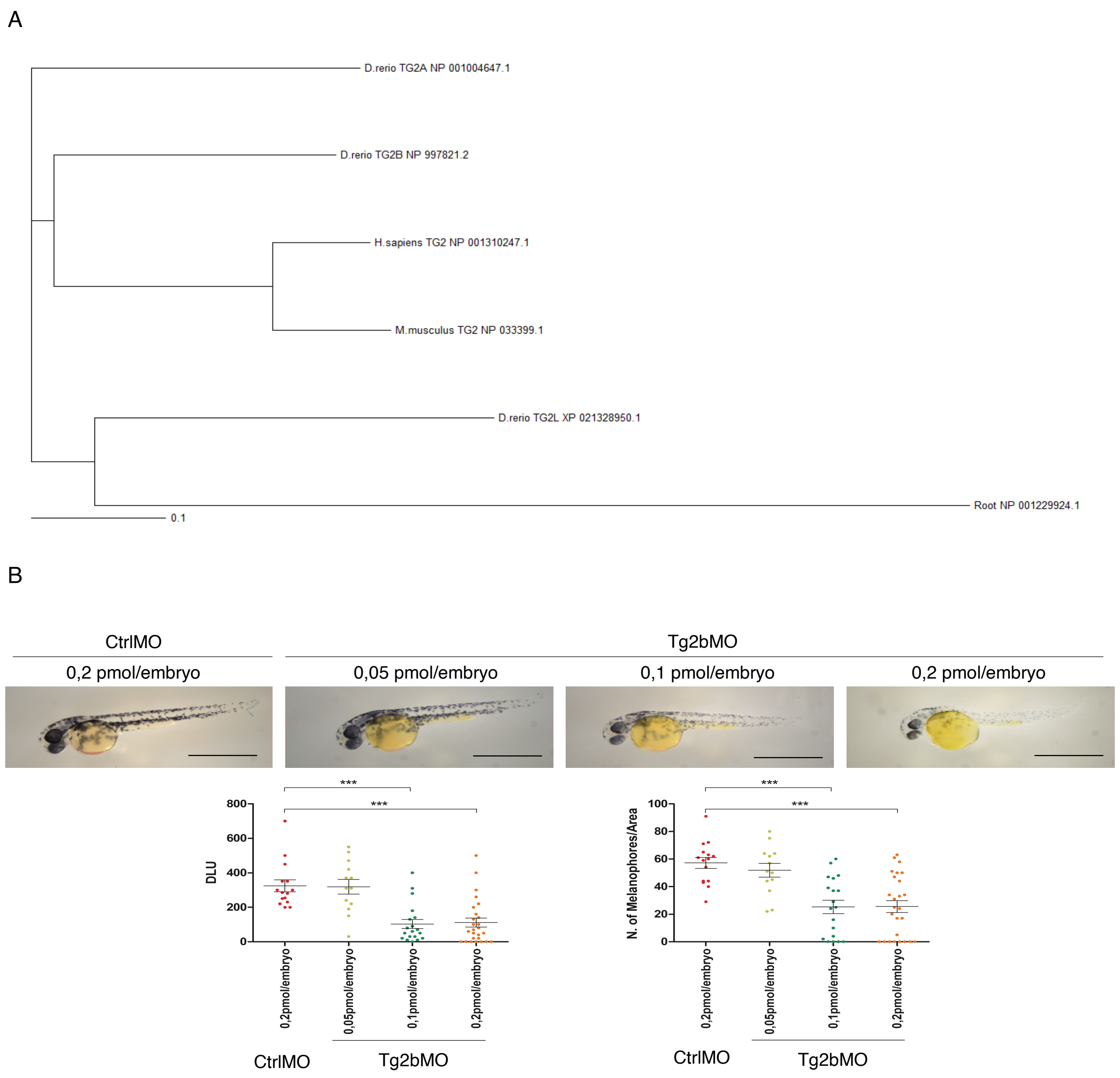

Supplementary Fig. S2 **A** N-J multialignment of *Danio rerio* TG2 paralogous proteins were compared and analyzed with murine and human TG2. Rooted phylogenetic cladogram is shown. Zebrafish zTg2b is the protein more similar to the human orthologous. **B** Knockdown of the zTg2b was performed by injecting three increasing dosages of morpholinos (0.05, 0.1 and 0.2 pmol). Pigmentation of embryos was analyzed by bright field microscopy. Graphs report the whole number of melanophores for each embryo (right panel) and the quantification of black pixels in the pictures (DLU, left panel) (means ± SEM, ***=p<0.001). Bars: 1mm.

A

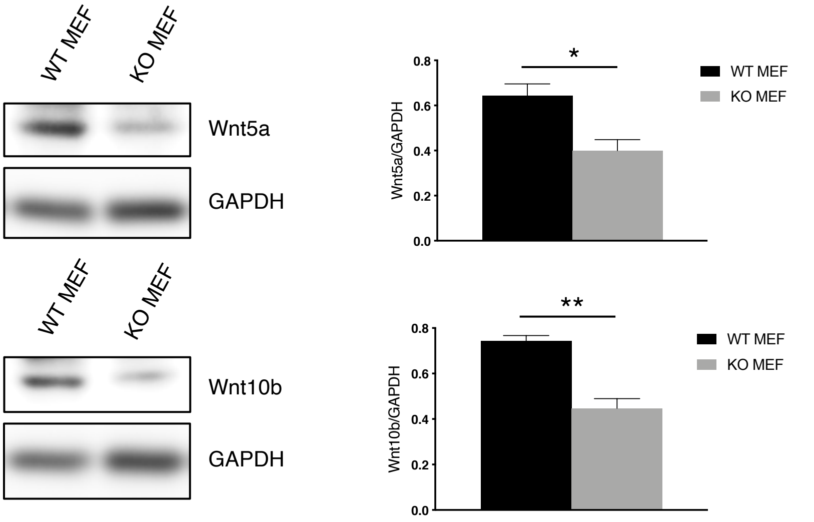

B

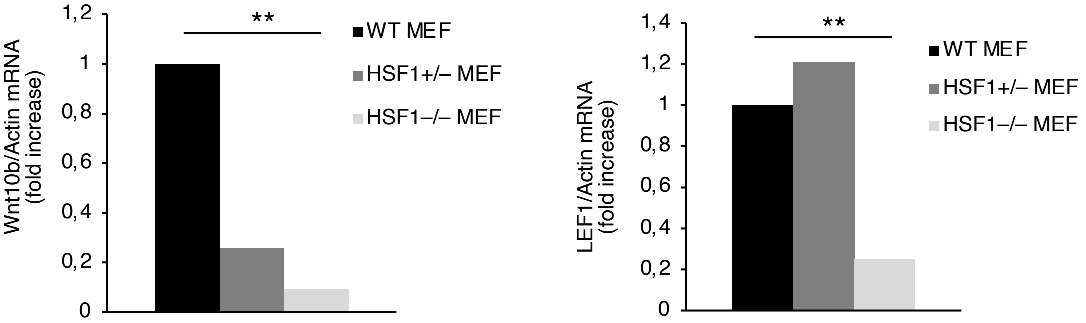

Supplementary Fig. S3 A Representative western blot and densitometric analysis of Wnt5a and Wnt10b in WT and KO MEFs. GAPDH was used as loading control (n =3; means ± SEM; *p<0.05; **p<0.01). **B** Wnt10b and LEF1 mRNA levels, quantified by qPCR, in WT, HSF1$^{+/-}$ and HSF1$^{-/-}$ MEFs. (n =3; means ± SEM; **p<0.01).

A

Protein IDs	Protein names	Gene names	Involvement in the WNT pathway
A6H6D1	Dapper homolog 1	Dact1	Regulates β-catenin activity (Yuan et al., 2012)
Q91ZS8	Double-stranded RNA-specific editase 1	Adarb1	Loss of Adarb1 induces altered expression in WNT targets (Qiu et al., 2013)
D3YYL1	B-cell CLL/lymphoma 7 protein family member B	Bcl7b	Negatively regulates the Wnt-signaling pathway (Uehara et al., 2015)
Q9JIX0	Transcription and mRNA export factor ENY2	Eny2	Inhibits WNT pathway (Nardi et al., 2018)
H3BJR8	Forkhead box protein P1	Foxp1	Inhibits/potentiates WNT and autophagy (Wang et al., 2019)
B1AQR8	Galectin	Lgals9	Involved in the WNT pathway (Salim et al., 2015)
A2ASR6	Polycomb complex protein BMI-1	Bmi1	Promotes cell proliferation and WNT/β-catenin signaling (Yu et al., 2018)
O35730	E3 ubiquitin-protein ligase RING1	Ring1	Activates the WNT pathway (Zhu et al., 2019)
A0A0G2JG60	SWI/SNF-related matrix-associated actin-dependent regulator of chromatin subfamily D member 3	Smarcd3	Induces the WNT5a (Jordan et al., 2013)
Q6P8X6	Putative ubiquitin carboxyl-terminal hydrolase 50	Usp50	
Q569U6	Transcription factor jun-B	Junb	
F8WGQ9	BTB/POZ domain-containing adapter for CUL3-mediated RhoA degradation protein 3	Kctd10	
Q9D8M4	60S ribosomal protein L7-like 1	Rpl7l1	
H3BJB6	T-complex protein 1 subunit theta	Cct8	
Q8BMA8	Elongation factor 2	Eef2	
Q8CBY8	Dynactin subunit 4	Dctn4	

B

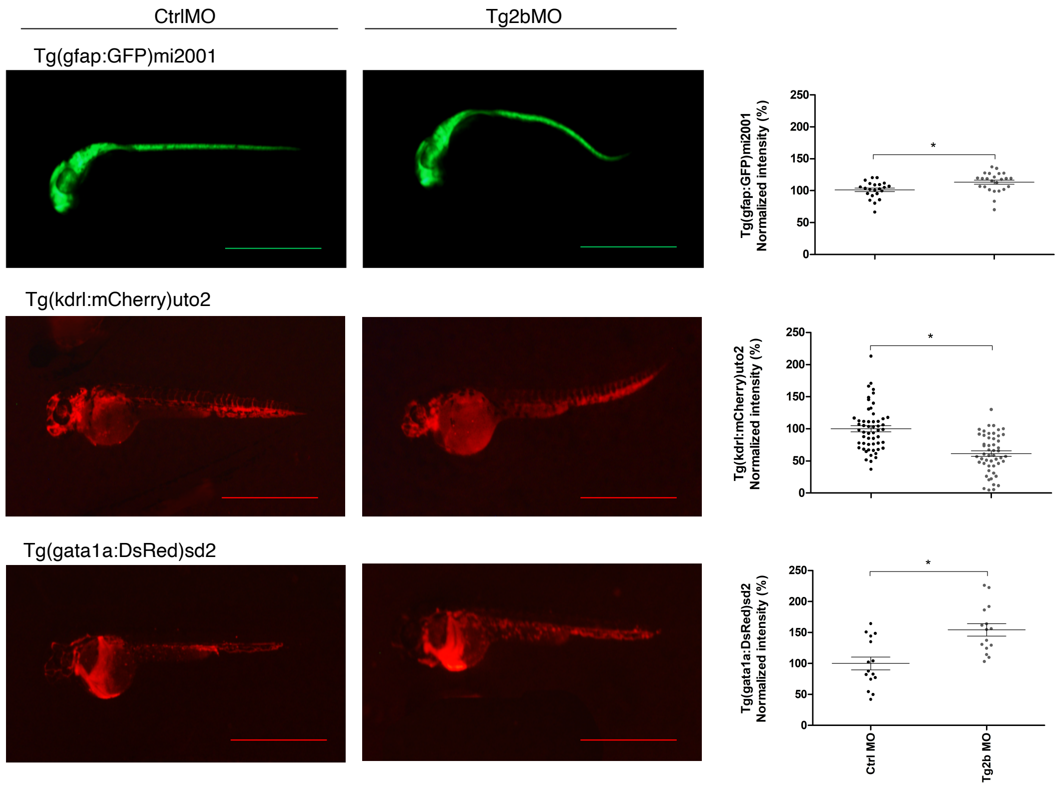

Supplementary Fig. S4 A List of the transcriptional factors identified in the TG2 interactome and characterization of the ones involved in the Wnt signaling. The protein complex containing TG2 and its interacting partners was obtained after immunoprecipitation of nuclear TG2 in WT MEFs, separation by HPLC and identification by mass spectrometry. **B** Knockdown of the zTg2b was performed in Tg(gfap:eGFP)mi2001, Tg(Kdrl:mCherry)uto2, Tg(Gata1:DsRed)sd2 and

compared to the control. The images are representative of different embryos. GFP epifluorescence quantification was reported in the graphs on the right. Dots represent treated embryos (means ± SEM; ∗=$p < 0.05$). Bars: 1 mm.
